# The complete plastome of *Echium plantagineum* L. (Boraginaceae), the first chloroplast genome belonging to the *Echium* genus

**DOI:** 10.1080/23802359.2022.2087559

**Published:** 2022-06-24

**Authors:** Inês Carvalho Leonardo, Maria Teresa Barreto Crespo, Jorge Capelo, Frédéric Bustos Gaspar

**Affiliations:** a iBET, Instituto de Biologia Experimental e Tecnológica, Oeiras, Portugal; b ITQB-NOVA, Instituto de Tecnologia Química e Biológica António Xavier, Universidade Nova de Lisboa, Oeiras, Portugal; c ECOCHANGE, CIBIO-InBIO - Research Centre in Biodiversity and Genetic Resources, Universidade do Porto, Vairão, Portugal; d INIAV, Instituto Nacional de Investigação Agrária e Veterinária I.P., Quinta do Marquês, Oeiras, Portugal

**Keywords:** *Echium plantagineum*, Boraginaceae, complete chloroplast genome, Illumina MiSeq sequencing, phylogenetic analysis

## Abstract

Besides being a common weed, the presence of *Echium plantagineum* L. in food and feed commodities can represent a safety hazard due to their content in pyrrolizidine alkaloids. In this study, the complete chloroplast of *E. plantagineum* isolate BPTPS251 is described, being the first available plastome from an isolate belonging to the *Echium* genus. The chloroplast genome is 149,776 bp in length with 37.5% GC content, displaying a quadripartite structure that contains a pair of inverted repeats regions (25,754 bp each), separated by a large single-copy (80,978 bp) and a small single-copy (17,290 bp) regions. A total of 131 genes were predicted, including 37 tRNA genes, 8 rRNA genes, and 86 protein-coding genes. The phylogenetic analysis confirmed the placement of *E. plantagineum* under the Boraginaceae family, belonging to the Boraginales order. This study will contribute to conservation, phylogenetic, and evolutionary studies, as well as DNA barcoding applications for food and feed safety purposes.

*Echium* L. (viper’s buglosses) is a genus of flowering plants (angiosperms) in the Boraginaceae family, including ca. 70 species distributed in North Africa, Continental Europe, Asia Minor, and Macaronesia Islands. Some viper’s buglosses were introduced elsewhere (Australia, South Africa, and the Americas), with some garden species (e.g., *Echium candicans* L.fil.) being found as alien escapes. Most species are annuals (e.g., *Echium vulgare* L.), rosette biennials (e.g., *Echium wildpretii* H.Pearson ex Hook.fil. in Tenerife, the Canaries), or seldom woody shrubs (e.g., *Echium vulcanorum* A.Chev. in Fogo Island, Cape Verde) (WFO 2022).

*Echium plantagineum* L. (1771) (Lady Campbell-weed) is a common weed in the European and Australian agricultural and waste ground areas (GBIF Secretariat 2021). Pharmacological activity of *Echium* include antimicrobial, antitumor, antiviral, and anti-inflammatory effects, mostly derived from shikonins found in the root (Wang et al. 2022). Seeds are also an important source of alpha-linolenic acid used in dietary supplements (Kitessa et al. 2011). Nectar of *E. plantagineum* may be important in late-winter honey production and as food for pollinators. It may also be hepatotoxic to cattle if chronically grazed due to the presence pyrrolizidine alkaloids (Moreira et al. 2020).

The Portuguese material of *E. plantagineum* analyzed, isolate BPTPS251, was collected from a wild population in Oeiras municipality in Portugal (Collection date: 20 May 2021; Location: 38.69846 N 9.31721 W) with a specimen being conserved at the LISE Herbarium (INIAV, Oeiras, Portugal; Jorge Capelo: jorge.capelo@iniav.pt) under the voucher LISE: 96329 (Identified by: Jorge Capelo).

Total genomic DNA was extracted from young leaves, frozen in liquid nitrogen immediately after collection and kept at −80 °C, using an adaptation of the Doyle and Doyle (1987) methodology. The obtained DNA was sheared by sonication using a Bioruptor (Diagenode), libraries were prepared with the NEBNext Ultra II DNA Library Prep Kit (New England Biolabs), and 150 bp paired-end sequencing was performed on an Illumina MiSeq platform using a v2 chemistry kit.

High-quality reads were used to assemble the complete chloroplast genome (sequence coverage: 582×) using the GetOrganelle pipeline (v1.7.5) (Jin et al. 2020), following the typical recipe suggested for Embryophyta plant plastome assembly (https://github.com/Kinggerm/GetOrganelle) with the additional option ‘-w 127’. The plastome annotation was performed using the GeSeq tool (Tillich et al. 2017) with a subsequent manual curation using Geneious Prime 2022.0.1 (https://www.geneious.com).

The chloroplast genome of *E. plantagineum* isolate BPTPS251 (GenBank accession number: OL335188) is 149,776 bp in length with 37.5% GC content, displaying a quadripartite structure that contains a pair of inverted repeat (IR) regions (25,754 bp, GC content 43.0%), separated by a large single-copy (LSC) region (80,978 bp, GC content 35.5%) and a small single-copy (SSC) region (17,290 bp, GC content 31.0%). A total of 131 genes were predicted, including 37 tRNA genes, 8 rRNA genes, and 86 protein-coding genes.

The phylogenetic analysis was performed using the concatenated sequences coding for the shared proteome extracted from all 11 verified and complete chloroplast genomes belonging to the Boraginales order available in GenBank (Accession date: 4 December 2021) and from the complete chloroplast genome of *E. plantagineum* obtained in this study. The sequences were aligned using MAFFT v7.450 (Katoh and Standley 2013) and further analyzed with the IQ-TREE 2 software package (Minh et al. 2020). The best-fit substitution model (GTR + F+R3 chosen according to the Bayesian Information Criterion) was selected according to ModelFinder (Kalyaanamoorthy et al. 2017), followed by a tree reconstruction (Figure 1) using IQ-TREE (Nguyen et al. 2015) using ultrafast bootstrap with UFBoot (10,000 replicates) (Hoang et al. 2018). The outgroup was *Salvia officinalis* L. (NC_038165) from the Lamiaceae family belonging to the Lamiales order.

**Figure 1.**
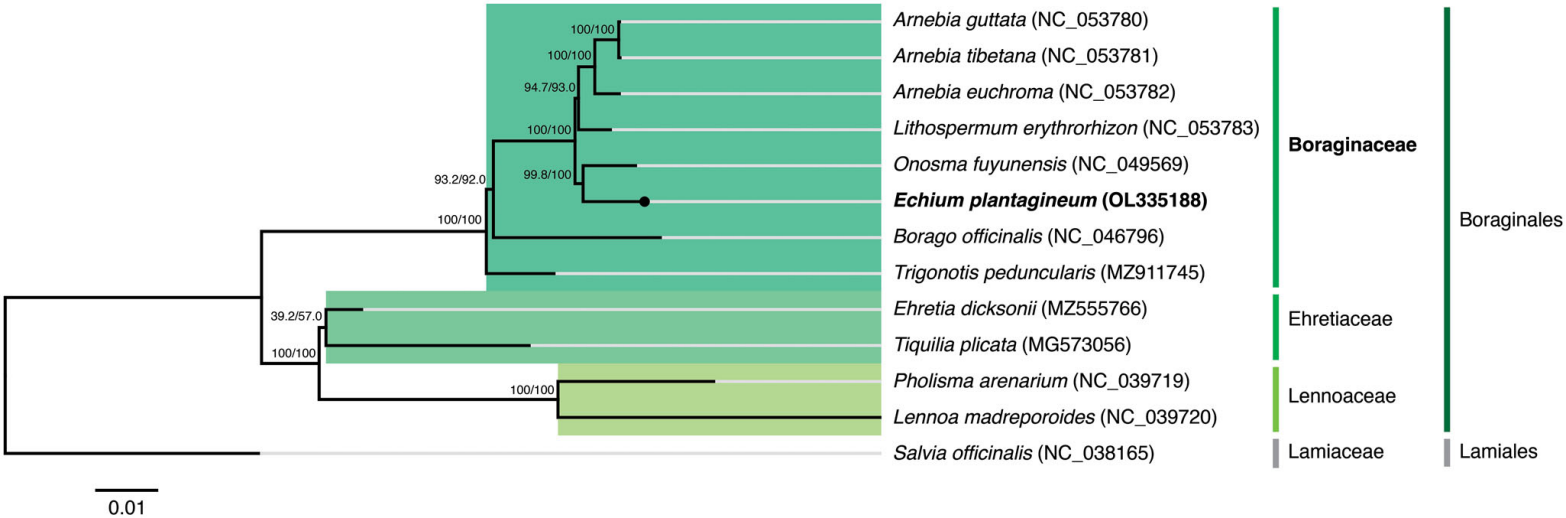
Maximum-likelihood tree inferred from the sequences coding for the shared proteome from *Echium plantagineum* isolate BPTPS251 and all 11 verified and complete chloroplast genomes belonging to the Boraginales order available in GenBank (Accession date: 2021.12.04). Numbers attached to the branches show the SH-aLRT and the UFBoot2 percent supports (SH-aLRT/UFBoot2). *Salvia officinalis* (Lamiales) was used as the outgroup.

The maximum likelihood tree showed that *E. plantagineum* is placed under the Boraginaceae family, belonging to the Boraginales order, and has a closer relationship with *Onosma fuyunensis* He & Liu with 99.8%/100% support (SH-aLRT/UFBoot2). The phylogenetic analysis performed with the alignments of the Boraginales complete chloroplast genomes also supports the same tree result.

This complete chloroplast genome will contribute to conservation, phylogenetic, and evolutionary studies, as well as DNA barcoding applications for food and feed safety purposes that target the detection of pyrrolizidine alkaloid-producing species.

## Data Availability

The data that supports this study is openly available in GenBank of NCBI at https://www.ncbi.nlm.nih.gov under the accession number OL335188. The associated BioProject, BioSample, and SRA numbers are PRJNA789447, SAMN22746547, and SRR16690175, respectively.
